# Trophic Nutrition in ICU Patients Undergoing High-Flow Oxygen Therapy and/or Noninvasive Mechanical Ventilation: The Nutri-Trophic Study

**DOI:** 10.3390/nu16091366

**Published:** 2024-04-30

**Authors:** Olivia Reta-Pérez, Manuel Colmenero-Ruiz, Carmen Rosa Hernández-Socorro, Pedro Saavedra, Silmary F. Maichle, Esther Portugal, Mariola Cerezo-Arias, Laura Sánchez Alés, Juan F. Martínez-Carmona, Lidon Mateu-Campos, Carol Lorencio-Cárdenas, Ana García-Miguélez, María Sosa-Durr, María San Martín-Bragado, Sergio Ruiz-Santana

**Affiliations:** 1 Department of Intensive Care, Hospital Universitario de Gran Canaria Dr. Negrín, Universidad de Las Palmas de Gran Canaria (ULPGC), Barranco de la Ballena s/n, 35010 Las Palmas de Gran Canaria, Spain; oretper@gobiernodecanarias.org (O.R.-P.); msosdur@gobiernodecanarias.org (M.S.-D.); msanbrae@gobiernodecanarias.org (M.S.M.-B.); 2 Department of Intensive Care, Hospital Universitario San Cecilio, A. del Conocimiento s/n, 18016 Granada, Spain; manuel.colmenero.sspa@juntadeandalucia.es; 3 Department of Radiology, Hospital Universitario de Gran Canaria Dr. Negrín, Universidad de Las Palmas de Gran Canaria (ULPGC), Barranco de la Ballena s/n, 35010 Las Palmas de Gran Canaria, Spain; chersoc@gobiernodecanarias.org; 4 Department of Mathematics, Universidad de Las Palmas de Gran Canaria (ULPGC), 35010 Las Palmas de Gran Canaria, Spain; pedro.saavedra@ulpgc.es; 5 Department of Intensive Care, Hospital Clínico Universitario San Carlos, Calle del Prof. Martín Lagos s/n, 28040 Madrid, Spain; silmaryfebrianny.maichle@salud.madrid.org; 6 Department of Intensive Care, Hospital Clínico Universitario de Valladolid, Av. Ramón y Cajal 3, 47003 Valladolid, Spain; esther_burgos@hotmail.com; 7 Department of Intensive Care, Hospital Universitario de Badajoz, Av.de Elvas s/n, 06080 Badajoz, Spain; maria.cerezo@salud-juntaex.es; 8 Department of Intensive Care, Hospital Universitari de Terrassa: CST, Carr. De Torrebonica s/n, 08227 Terrassa, Spain; lsancheza@cst.cat; 9 Department of Intensive Care, Hospital Regional Universitario de Málaga, Av. De Carlos Haya 84, 29010 Málaga, Spain; jfrancisco.martinez.sspa@juntadeandalucia.es; 10 Department of Intensive Care, Hospital General Universitario de Castellón, Avinguda de Benicàssim 128, 12004 Castelló de la Plana, Spain; mateuli@hotmail.com; 11 Department of Intensive Care, Hospital Universitari Dr Josep Trueta, Avinguda de França, s/n, 17007 Girona, Spain; carol_lorencio@hotmail.com; 12 Department of Intensive Care, Hospital Universitario Marqués de Valdecilla, Av. de Valdecilla s/n, 39008 Santander, Spain; ana.garciam@scsalud.es

**Keywords:** acute respiratory failure, high-flow nasal oxygen therapy, noninvasive ventilation, enteral nutrition critically, trophic feeding, nutrition support

## Abstract

Enteral nutrition (EN) therapy in ICU patients requiring oxygen therapy with high-flow nasal cannula (HFNC) and/or noninvasive mechanical ventilation (NIMV) is controversial. A prospective, cohort, observational, and multicenter study was conducted in 10 ICUs in Spain to analyze the 90-day mortality, tolerance, side effects, and infectious complications of trophic EN in patients requiring HFNC therapy and/or NIVM. A total of 149 patients were enrolled. The mean age, severity scores, tracheobronchitis, bacteremia, and antimicrobial therapy were significantly higher in deceased than in living patients (*p* < 0.05), and the mortality rate was 14.8%. A total of 110 patients received oral trophic feedings, 36 patients received nasogastric tube feedings (NGFs), and 3 received mixed feedings. Trophic EN was discontinued in only ten (14.9%) patients because of feeding-related complications. The variables selected for the multivariate logistic regression on feeding discontinuation were SOFA upon admission (OR per unit = 1.461) and urea (OR per mg/dL = 1.029). There were no significant differences in the development of new infections according to the route of EN administration. Early trophic feeding administered to patients with acute respiratory failure requiring noninvasive ventilation is safe and feasible, and is associated with few dietary and infectious complications in a mortality, setting comparable to similar studies.

## 1. Introduction

The ventilation and oxygenation of patients, especially in intensive care units (ICUs), are important therapeutic tools for patient management [1,2]. Among the main ventilation and oxygenation methods available to us are noninvasive mechanical ventilation (NIVM) with face mask and high-flow nasal cannula (HFNC) [1,2,3,4,5,6]. NIVM has represented a treatment option in patients with acute respiratory failure before intubation or reintubation is considered [7]. HFNC ventilation has offered an advance in the oxygenation of patients in acute respiratory failure, avoiding intubation, and has also been a resource that allows intubated patients on mechanical ventilation to be disconnected [5]. Among the well-studied side effects that can arise with these two noninvasive oxygenation mechanisms are bronchial aspiration, gastric insufflation, aerophagia and sialorrhea.

High-flow ventilation consists in increasing the gas mixture by releasing high flows of oxygen and air, in modifiable proportions, in such a way that positive pressures are achieved in the airway, facilitating the entry of this gas during spontaneous ventilation [8], with better oxygenation results than conventional oxygen therapy methods [7]. This increase in positive pressure could be a facilitating element for digestive intolerance, either by swallowing air and gastric distension, or by causing incontinence of the esophageal sphincters and thus facilitating regurgitation and broncho-aspiration of gastric contents. On the other hand, NIVM consists of ventilatory support applied without the use of endotracheal or pharyngeal devices, increasing alveolar ventilation by applying positive pressure throughout the airway via an interface that acts on the airway pressure gradient to maintain adequate gas exchange [3,5,6]. This increase in positive pressure, similar to ventilation with HFNC, could also be an element favoring digestive intolerance.

Patients undergoing respiratory failure often present high metabolic stress leading to a hypercatabolic situation, and may be unable to feed for days, increasing the risk of malnutrition or worsening pre-existing malnutrition. This situation is associated with various complications, thus increasing morbidity and mortality, hospital stay, and costs [8,9,10]. The nutritional risk that determines this situation is high, so nutritional therapy is justified. This nutritional therapy in spontaneously ventilated patients is usually complemented with oral feeding, but it is not so easy for them to receive and tolerate adequate levels of caloric and protein intakes.

The method of choice for nutritional therapy in patients at high nutritional risk would be, if possible, the oral route or, failing that, enteral nutrition (EN) via the nasogastric feeding (NGF) route. This is due to the advantages it will provide for the patient’s health by maintaining the digestive tract in a functional state [11,12]. Therefore, the administration, through early initiation, of an oral or nasogastric diet helps to prevent intestinal villous atrophy, enterocyte apoptosis, inflammatory infiltration, dysbiosis, and impaired intestinal immune functions [13]. The possibility of administering enteral nutrition may alleviate or even reverse some of these pathophysiological cascades [11,12,13,14,15,16]. 

Clinical data also support early EN, between 24 and 48 h after ICU admission, in ICU patients [17]. Several meta-analyses of randomized–controlled trials have demonstrated that early EN, compared with delayed EN, was associated with lower infectious morbidity in patients admitted to the ICU [18,19,20]. In addition, the energy intake in the early phase (4–7 days) should be lower than the energy expenditure, and then increased to match energy expenditure later to avoid overfeeding [21]. Furthermore, there is no evidence of increased protein intake in prospective randomized trials [22,23,24,25] by critically ill patients in terms of clinically relevant outcomes. However, it has recently been reported that the administration of higher doses of protein in mechanically ventilated ICU patients did not improve the time to hospital discharge and, moreover, could worsen outcomes, particularly in patients with acute kidney injury and elevated organ failure scores [26,27,28,29]. 

The decision to initiate nutritional therapy, either orally or by nasogastric tube, in critically ill patients requiring oxygen therapy with HFNC and/or NIV is currently a subject of debate. Despite the benefits associated with this practice in these patients, the scarcity of clinical studies with sufficient methodological quality, as well as the lack of specific recommendations on oral/enteral NGF nutritional therapy, has generated controversy among professionals involved in the care of ICU patients. In fact, this topic is not addressed in the latest published guidelines on nutritional support in the ICU [13,21].

The main objective of our study was to evaluate mortality at 90 days in well-nourished ICU patients requiring HFNC therapy and/or NIMV, and who received trophic nutrition with a hyperproteic diet administered orally or by enteral NGFs. The secondary objective was to evaluate the tolerance, safety, and infectious complications of these administrations.

## 2. Materials and Methods

### 2.1. Study Design (Figure 1)

This was a prospective, observational, and multicenter study that analyzed the 90-day mortality, tolerance, and side effects of trophic enteral nutrition, administered either orally or by NGF, as well as infectious complications, in a cohort of patients requiring HFNC oxygen therapy and/or NIVM who were admitted to ten Spanish ICUs between January 2019 to August 2023.

HFNC/NIMV indications. The type of respiratory support will depend on the investigators’ choice [3,4,5,6,7]:

- Acute respiratory failure, including exacerbation of chronic obstructive pulmonary disease (COPD), in postoperative, immunocompromised patients;
- Prevention of acute respiratory failure in high-risk postextubation patients and initial management of these patients;
- Acute heart failure;
- Obstructive sleep apnea syndrome;
- Thoracic trauma.

Inclusion criteria:

- Age ≥ 18 years;
- Signed informed consent;
- Requirement of HFNC oxygen therapy and/or NIMV;
- Duration of HFNC/NIMV or oxygen therapy ≥ 24 h;
- Expected survival > 72 h;
- ICU stay ≥ 72 h.

Exclusion criteria:

- Body mass index (BMI) < 18;
- No requirement of HFNC oxygen therapy and/or NIMV;
- Absolute contraindication to trophic EN (active gastrointestinal bleeding, intestinal obstruction, etc.) or nonfunctioning gastrointestinal tract.

**Figure 1:**
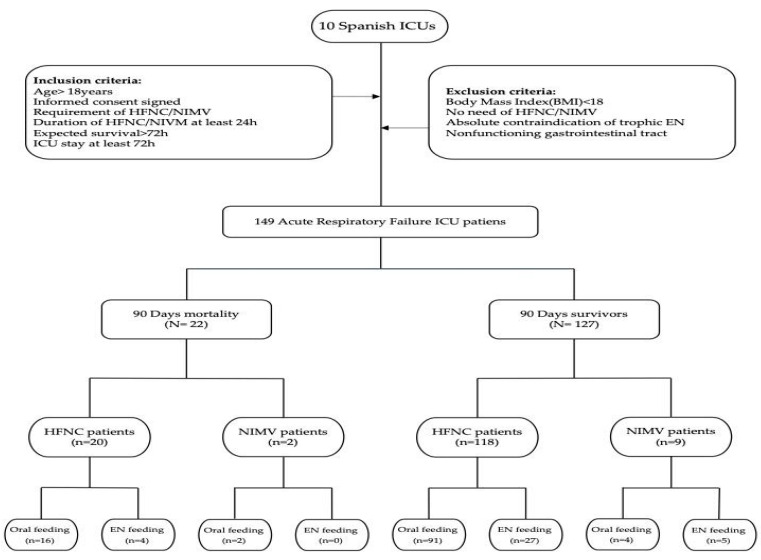
HFNC: high-flow nasal cannulae; NIMV: noninvasive mechanical ventilation; EN: enteral nutrition.

### 2.2. Trophic Nutrition

Nutritional treatment was administered in the form of trophic nutrition according to a protocol established in each unit and known to the medical and nursing staff. As a guideline, information on the protocol used in previous studies by the Metabolism and Nutrition Working Group was provided [16]. Nutritional treatment was administered on the basis of the defined trophic quantity (energy target of 20–30% of the estimated caloric needs of 20–30 kcal/kg [23] and a protein intake of 1.2 to 2.0 g/kg/day, to be achieved within 72 h of the initiation of nutritional therapy) [30,31,32,33]. The route of access for enteral nutritional support was either orally or by NGFs, for which in the latter case the diet could be flavored with coffee powders without caffeine.

The rate of initiation and augmentation of the oral/NGF intakes were at the discretion of each participating ICU. Prokinetics and parenteral nutrition (PN) were not to be used routinely, and their indication was at the discretion of the attending physician [22,34]. A hyperproteic nutritional formula (10 g/100 mL) was used, with a caloric intake of 1.2 kcal/mL and a nonprotein calories/nitrogen ratio of 52:1 (Fresubin Intensive, Fresenius-kabi, Germany).

Figure 1 shows the design and flow diagram of the study. 

Trophic NE was administered for 23 h each day using a continuous infusion pump or orally, upon patient request, until the daily target was reached. The patients’ beds were elevated, as much as possible, to greater than 30° to reduce the risk of aspiration. The gastric residual volume (GRV) was measured every 24 h [16] in patients receiving enteral nutrition via NGFs. We maintained trophic feeding for no more than 7 days after its initiation [22,25]. The duration of observation of total energy intake was until we discontinued trophic nutritional feeding, after admission to the ICU.

A list of standardized definitions of the possible complications related to EN was also established. Thus, an increase in gastric residual values was defined as a GRV obtained at each assessment of greater than 500 mL [16,21,26]. Abdominal distension was defined as a change in abdominal circumference detected by physical examination compared with the clinical assessment prior to the onset of EN. Regurgitation was defined as the presence of EN in the oral or oropharyngeal cavity, as well as its spontaneous drainage orally or by NGF. Diarrhea associated with NE was defined as the presence of 5 or more liquid stools within 24 h or more than 2 stools of 1000 mL each in a 24 h interval. Constipation was defined as the absence of bowel movements for 5 days from the onset of NE or for a period of 3 days from the first week of admission. Aspiration was defined as the presence of respiratory secretions with characteristics similar to the nutrition administered. All complications described were evaluated daily by the responsible physician and recorded on the data collection sheets.

### 2.3. Study Variables

(a) Patients general characteristics:

- Age and sex;
- Weight in kg and height in meters. Body mass index (BMI) in kg/m^2^;
- Severity scales—Apache-II [18] (first 24 h);
- Admission reasons/patients type—sepsis, cardiac surgery, other surgeries, nonsurgical cardiology, trauma, burns, pneumonia (community-acquired, healthcare-associated, or hospital-acquired), acute respiratory distress syndrome (ARDS), and others;
- Number of days NIMV and/or HFNC therapy received. Number of days ICU and hospital stay.
- 90-day mortality.

(b) Other variables:

- Scores for the Sequential Organ Failure Assessment (SOFA) scale [20]—days 0 and 3;
- Nosocomial infections (tracheobronchitis and ventilator-associated pneumonia (VAP), bacteremia, and urinary tract infections (UTIs)) defined according to the ENVIN-HELICS) criteria [35].
- For other infections, we recorded (if applicable):
- Presence of continuous renal replacement therapy (CRRT);
- Patients receiving acute mucosal gastro-duodenal lesions (AMGDLs) prophylaxis;
- Levels of albumin, prealbumin, retinol, and transferrin upon admission and weekly;
- Maximum bilirubin, AST, ALT, ALP, and GGT levels;
- Patients receiving antimicrobial treatment.

(c) Variables recorded related to trophic EN:

- Number of days of EN with HFNC/VMNI;
- Energy target (kcal);
- Enteral volume administered per day (mL);
- Nutritional calories received per day (kcal/kg/day);
- Enteral caloric intake per day (kcal/kg/day);
- Total caloric intake, all sources, per day (kcal/kg/day);
- Ratio of received calories (%) to target calories (kcal/kg/day);
- Parenteral dextrose intake per day (kcal/kg/day);
- Median (interquartile range (IQR)), minimum and maximum blood glucose per day;
- Patients receiving prokinetics;
- Energy balance;
- Prescribed protein (g/day);
- Daily protein intake (g/kg/day);
- Ratio of received protein (%) to target protein (g/day).

(d) Safety recorded variables:

- Gastric residual (≥ 500 mL/day);
- Abdominal distension;
- Diarrhea;
- Vomiting/regurgitation;
- Broncho-aspiration;
- Nasogastric tube complications;
- Discontinuance/reason trophic EN.

### 2.4. Outcomes of the Study

We consider as outcomes of the study the following binary variables:

- Death (90-day mortality);
- Discontinuation of nutritional therapy;
- Infectious complications.

### 2.5. Statistical Analysis

#### 2.5.1. Design

This was a prospective observational study in which, for each patient recruited, the sets of daily and weekly observations corresponding to each marker were summarized as medians. The following definitions were used:

- Ratio of received calories (%) to target calories (kcal/kg/day) = (delivered enteral kcal + delivered parenteral kcal / target kcal);
- Ratio of received protein (%) to target protein (g/day) = (gr. delivered enteral proteins + gr. delivered parenteral proteins/grs. target proteins);
- A patient was considered to have infectious complications if and only if he/she presented at least one of the following events: tracheobronchitis, VAP, bacteremia, UTI, or any other infection.

#### 2.5.2. Sample Size Calculation

Data from a pilot study showed that the 90-day mortality rate could reach between 10% and 11%. Assuming a rate of 11%, the sample size required to estimate the rate with an error rate of 5% and 95% confidence was *n* = 150.

#### 2.5.3. Univariate Analysis

The association of each outcome with the study variables described above was performed using univariate analysis. For this purpose, in each of the groups defined by the outcome, categorical variables are expressed as frequencies and percentages, and continuous variables as the mean and standard deviation (SD), when the data follow a normal distribution, or as the median and interquartile range (IQR = 25th–75th percentile) when the distribution deviates from normality. Percentages were compared, if appropriate, using the chi-square test or Fisher’s exact test, means with the t-test, and medians with the Wilcoxon test for independent data.

#### 2.5.4. Multivariate Logistic Regression

For each of the outcomes, a multivariate logistic analysis was performed. Variables that showed a significant association with the outcome in the univariate analysis underwent a multivariate analysis. The selection of variables was based on the best subset regression and Bayesian information criterion (BIC). The model is summarized using *p*-values (likelihood ratio test) and odds ratios, which were estimated by means of 95% confidence intervals. Statistical significance was set at *p* < 0.05. Data were analyzed using the R package, version 4.2.1 (R Development Core Team, 2022 [36]).

## 3. Results

### 3.1. Demographic and General Data According to Survival (Table 1)

A total of 149 patients were enrolled, with 110 receiving only oral trophic feedings, 36 receiving trophic NGFs, and 3 receiving mixed feedings. The reasons for admission were as follows: community-acquired pneumonia, 68; hospital-acquired pneumonia, 4; ARDS, 23; sepsis, 18; trauma, 11; postoperative cardiac surgery, 5; nonsurgical cardiac, 3; other, 17. One hundred and thirty-eight patients required HFNC therapy and the remaining eleven required NIMV. The median number of days for which patients required HFNC therapy was three, while that for NIVM was four, and there were no significant differences between both groups studied (*p* = 1). The median age was 62.6 years, and 68.5% of patients were male. The median body mass index (BMI) was 28.1, and the glycemic data and levels of albumin, prealbumin, and retinol upon admission were normal, showing no significant differences among the groups studied.

The median Apache-II score was 15, but the scores were significantly higher in the nonsurvivors group (*p* = 0.001). The median SOFA score was 4 upon admission and 3 on the third day, with the latter measurement being significantly higher in the nonsurvivors group (*p* < 0.001).

The median numbers of days of ICU and hospital stays were 9 and 15, respectively. ICU days of stay were almost significantly higher for deceased patients versus those who survived (*p* = 0.06). Eight percent of patients required continuous renal replacement therapy (CRRT). The use of CRRT was significantly higher in the deceased versus the surviving group (*p* < 0.001). In addition, the discontinuance of trophic enteral nutrition was not required in the patients undergoing CRRT. Daily therapeutic nutritional data are shown in all of the tables. There were no differences in the glycemic data among all groups studied.

**Table 1:** Patient characteristics according to survival.

	Overall N = 149	Survivors N = 127	Nonsurvivors N = 22	*p*-Value
Age (years)	62.6 ± 13.9	61.6 ± 14.0	68.2 ± 12.7	0.041
Sex male	102 (68.5)	88 (69.3)	14 (63.6)	0.598
Body mass index (kg/m^2^)	28.1 ± 5.6	28.3 ± 5.5	27.1 ± 5.8	0.359
Apache-II score	15 (10; 20)	14 (19; 19)	18 (15; 22)	0.01
SOFA upon admission	4 (2; 6)	3 (2; 6)	6 (3; 8)	0.015
SOFA on day three	3 (2; 6)	3 (2; 5)	6 (3; 7)	0.008
Complications				
Tracheobronchitis	12 (8.1)	7 (5.5)	5 (22.7)	0.018
VAP	10 (6.7)	7 (5.5)	3 (13.6)	0.168
Bacteremia	12 (8.1)	5 (3.9)	7 (31.8)	<0.001
UTIs	9 (6.0)	4 (3.1)	5 (22.7)	0.004
Other infections	9 (6.0)	6 (4.7)	3 (13.6)	0.13
CRRT	12 (8.1)	5 (3.9)	7 (31.8)	<0.001
Prophylaxis AGDML	129 (86.6)	109 (85.8)	20 (90.9)	0.739
Prokinetics	22 (14.8)	16 (12.6)	6 (27.31)	0.099
Antimicrobial treatments	81 (54.4)	61 (48.0)	20 (90.9)	<0.001
90-Day mortality	22 (14.8)	0	22 (100.0)	<0.001
Oral feedings	110 (76.9)	92 (72.4)	18 (81.8)	0.467
NGFs	36 (25.2)	29 (22.8)	7 (31.8)	0.72
Diarrhea	18 (12.1)	16 (12.6)	2 (9.1)	1
Gastric residue > 500 mL (n)	2 (1.3)	2 (1.6)	0	1
Vomiting/regurgitation	1 (0.7)	1 (0.8)	0	1
Broncho-aspiration	0	0	0	1
NG tube obstruction	1 (0.7)	1 (0.8)	0	1
Abdominal distention	4 (2.7)	2 (1.6)	2 (9.1)	0.104
NG tube displacement	4 (2.7)	4 (3.1)	0	1
EN discontinuation	10 (6.7)	7 (5.5)	3 (13.6)	0.168
Oxygen therapy type:				0.666
HFNC	138 (92.6)	118 (92.9)	20 (90.9)	
NIMV	11 (7.4)	9 (7.1)	2 (9.1)	
ICU days	9 (6; 16)	9 (6; 14)	16 (7; 30)	0.019
Hospital days	15 (8; 23)	16 (10; 23)	14 (7; 26)	0.802
HFNC days	3 (2; 4)	3 (2; 4)	2 (2; 3)	0.064
NIMV days	4 (3; 6)	4 (3; 6)	4 (3; 4)	0.158
Albumin (g/dL)	3 (3; 3)	3 (3; 3)	3 (3; 3)	0.116
Prealbumin (mg/dL)	15 (10; 20)	15 (10; 21)	15 (8; 18)	0.558
Retinol (UI)	4 (2; 5)	4 (2; 5)	4 (3; 6)	0.296
Transferrin (md/dL)	154 (122; 176)	158 (127; 177)	117 (97; 130)	0.009
Bilirubin (mg/dL)	0.53 (0.35; 0.78)	0.49 (0.35; 0.68)	0.80 (0.52; 1.19)	0.007
AST (U/L)	38 (21; 54)	38 (22; 52)	36 (18; 76)	0.896
ALT (U/L)	37 (20; 58)	38 (20; 60)	26 (15; 55)	0.225
GGT (U/L)	79 (60; 108)	73 (38; 138)	62 (52; 130)	0.977
ALP (U/L)	69 (39; 138)	79 (60; 102)	82 (61; 121)	0.707
INR	1.10 (1.01; 1.19)	1.10 (1.00; 1.20)	1.12 (1.06; 1.17)	0.603
Prothrombin (s)	13 (12; 15)	13 (12; 15)	13 (12; 14)	0.447
Urea (mg/dL)	52 (39; 74)	50 (38; 67)	74 (51; 89)	0.009
Creatinine (mg/dL)	0.79 (0.61; 1.17)	0.77 (0.61; 1.04)	1.23 (0.70; 1.47)	0.042
Daily data *				
Energy target (Kcal)	1811 (1500; 2180)	1825 (1500; 2150)	1775 (1470; 2240)	0.696
Volume of enteral administration (mL)	450 (267; 500)	500 (288; 500)	335 (228; 438)	0.01
Enteral intake (Kcal)	520 (300; 600)	549 (300; 600)	396 (277; 480)	0.02
Ratio of energy intake/target	0.28 (0.18; 0.36)	0.29 (0.19; 0.38)	0.24 (0.17; 0.32)	0.208
Parenteral dextrose intake (Kcal)	70 (0; 190)	46 (0; 180)	150 (101; 200)	0.007
Prescribed protein (g/day)	50 (50; 90)	50 (50; 92)	50 (49; 50)	0.051
Protein intake (g/day)	50 (25; 50)	50 (25; 50)	33 (23; 48)	0.028
Ratio of protein intake/target	0.81 (0.49; 1.00)	0.90 (0.49; 1.00)	0.65 (0.51; 0.85)	0.265
Gastric residue (mL) (n)	69 (50; 128) (n = 26)	62 (50; 108) (n = 24)	180 (150; 210) (n = 2)	0.101
Total kcal intake	600 (449; 750)	600 (458; 755)	571 (397; 705)	0.281
Caloric intake (Kcal/kg)	7 (5; 10)	7 (5; 10)	7 (6; 9)	0.83
Ratio of total energy intake/target	0.32 (0.23; 0.44)	0.31 (0.23; 0.45)	0.34 (0.24; 0.42)	0.944
PN kcal intake (n)	1322 (439; 1568) (n = 6)	1518 (1125; 1585) (n = 5)	160 (160; 160) (n = 1)	0.143
PN protein intake (g) (n)	84 (76; 96) (n = 6)	83 (74; 99) (n = 5)	84 (84; 84) (n = 1)	0.77
Propofol kcal intake (n)	82 (18; 143) (n = 4)	82 (18; 143) (n = 4)	(n = 0)	
Glycemia (mg/dL)				
Median	130 (113; 153)	130 (112; 152)	134 (123; 161)	0.378
Minimum	110 (93; 128)	110 (94; 127)	113 (93; 131)	0.522
Maximum	153 (130; 189)	150 (126; 184)	176 (140; 228)	0.077

* Daily data for each patient are summarized as the medians. Data are presented as the means ± SD, frequencies (%), and medians (IQR). Apache: acute physiology and chronic health assessment. SOFA: sequential organ failure assessment. VAP: ventilator-associated pneumonia. UTIs: urinary tract infections. CRRT: continuous renal replacement therapy. NG: nasogastric. NGF: nasogastric tube feeding. EN: enteral nutrition. HFNC: high-flow nasal cannulae. AST: aspartate aminotransferase. ALT: alanine aminotransferase. GGT: gamma glutamyl transpeptidase. ALP: alkaline phosphatase. INR: international normalized ratio. PN: parenteral nutrition.

### 3.2. Mortality (Table 1)

Twenty-two patients (14.8%) died within 90 days of admission to ICU. There were significantly lower volumes and kilocalories administered among the deceased compared to the survivors (*p* < 0.05). However, the nonsurvivors received significantly less protein than the survivors (*p* < 0.01). Episodes of feeding intolerance were rare, but feeding interruptions were nonsignificantly more frequent for the deceased than living patients. None experienced clinically demonstrated episodes of aspiration, but 14.8% received prokinetics. Tracheobronchitis, urinary tract infections (UTIs), and antibiotic treatment were significantly higher in nonsurvivors vs. survivors (*p* < 0.05).

One hundred and ten patients received oral trophic feedings, thirty-six received trophic NGFs and three received mixed feedings, and there were no significant differences in mortality. The median (IQR) prescribed protein intake was significantly higher for survivors than deceased patients (*p* < 0.05).

One hundred and thirty-eight received HFNC therapy and the remaining eleven received NIVM. The median number of days for which patients received HFNC therapy was 3, while this number was 4 for NIVM; there were no significant differences among the groups studied (*p* = 1). Propofol was administered to four patients, all of whom survived. In addition, six patients received parenteral nutrition, five of whom survived. Seventeen patients were eventually mechanically ventilated, and eight did not survive. In addition, among these eight non-survivors, seven were ventilated after HFNC treatment and one after NIMV.

According to results of the univariate analysis, the variables included in the multivariate logistic analysis for “Death” were: age (years), Apache-II score, SOFA at admission, SOFA on third day, tracheobronchitis, bacteremia, CRRT, antimicrobial treatments, transferrin (md/dL), albumin (g/dL), bilirubin (mg/dL), urea (mg/dL), creatinine (mg/dL), prescribed protein_g/day, protein intake_g/day.

As shown in Table 2, the variables selected for the multivariate logistic regression on survival were age (OR per year = 1.068; 95% CI = 1.020–1.117), bacteremia (OR = 7.013; 95% CI = 1.341–36.7), and CRRT (OR = 20.3; 95% CI = 3.78–109).

### 3.3. Nutrition-Related Data, Outcomes, and Discontinuance of Nutritional Therapy (Table S1)

In total, 30 patients (20.1%) had at least one feeding-related complication, 18 of whom had diarrhea or, more rarely, abdominal distension (n = 4), displacement of the nasogastric tube (n = 4), increased gastric residual to greater than 500 mL (n = 2), or vomiting (n = 1). Vomiting and regurgitations were infrequent, and there were no episodes of broncho-aspiration. Trophic enteral nutrition had to be discontinued because of feeding-related complications in ten (14.9%) patients. In 5 of the 119 patients with no diet-related complications, the diet was eventually discontinued as they refused to drink it because they disliked the taste.

According to the results of the univariate analysis for “discontinuation of nutritional therapy”, the following variables were included in the multivariate logistic analysis: Apache-II score, SOFA at admission, SOFA on third day, diarrhea, abdominal distention, ALP(U/L), urea (mg/dL), and creatinine (mg/dL).

As shown in Table 3, the variables selected for the multivariate logistic regression on the discontinuation of nutrition administered orally or by NGFs were SOFA upon admission (OR per unit = 1.461; 95% CI = 1.051–2.032) and urea (OR per mg/dL = 1.029; 95% CI = 1.013–1.045).

### 3.4. Infectious Complications (Table 4)

Mortality at 90 days was significantly higher (*p* < 0.01) among patients with infectious complications than for those without. Apache-II and SOFA scores on day 3 and antibiotic use were significantly higher for patients with at least one infectious complication (*p* < 0.05). One hundred and ten patients received oral trophic feedings, thirty-six received nasogastric tube feedings and three received mixed feedings, and there were no significant differences in the infectious complications. Propofol was administered to four patients, and two of them had infectious complications. In addition, six patients received parenteral nutrition, and only one of them had infectious complications.

In total, 138 received HFNC therapy and the remaining 11 received NIVM. The median numbers of days were three for HFNC therapy and four for NIVM, and there were no significant differences among both groups studied. Twelve patients received CRRT, and eight of them had at least one infectious complication, which is significantly higher compared to those without (*p* < 0.005). The use of prokinetics was also significantly higher in those patients with at least one infectious complication (*p* < 0.05).

The median numbers of days of ICU and hospital stays were 9 and 15, respectively. Days of ICU stay were significantly higher in patients with at least one infectious complication versus those without (*p* < 0.001).

**Table 4:** Patient characteristics overall and according to infectious complications.

	Infectious Complications		
	No N = 112	Yes N = 37	*p*-Value
Age (years)	62.5 ± 13.8	62.8 ± 14.5	0.924
Sex male	74 (66.1)	28 (75.7)	0.276
Body mass index (Kg/m^2^)	28.1 ± 5.8	28.3 ± 4.9	0.885
Apache-II score	14 (10; 19)	17 (12; 21)	0.046
SOFA upon admission	3 (2; 6)	4 (3; 7)	0.128
SOFA on day 3	3 (2; 5)	4 (3; 7)	0.013
Complications			
Tracheobronchitis	0	12 (32.4)	<0.001
VAP	0	10 (27.0)	<0.001
Bacteremia	0	12 (32.4)	<0.001
UTIs	0	9 (24.3)	<0.001
Other infections	0	9 (24.3)	<0.001
CRRT	4 (3.6)	8 (21.6)	0.002
Prophylaxis_AGDML	94 (83.9)	35 (94.6)	0.162
Prokinetics	12 (10.7)	10 (27.0)	0.015
Antimicrobial treatments	48 (42.9)	33 (89.2)	<0.001
90-day mortality	8 (7.1)	14 (37.8)	<0.001
Oral feedings	83 (76.8)	27 (77.1)	0.972
NGFs	27 (25.0)	9 (25.7)	0.933
Diarrhea	15 (13.4)	3 (8.1)	0.563
Gastric residue > 500 mL (n)	2 (1.8)	0	1
Vomiting/regurgitation	0	1 (2.7)	0.248
Broncho-aspiration	0	0	1
NG tube obstruction	0	1 (2.7)	0.248
Abdominal distention	4 (3.6)	0	0.572
NG tube displacement	2 (1.8)	2 (5.4)	0.257
EN discontinuation	9 (8.0)	1 (2.7)	0.452
Oxygen therapy type:			0.467
HFNC	105 (93.8)	33 (89.2)	
NIMV	7 (6.2)	4 (10.8)	
ICU days	8 (6; 14)	12 (9; 24)	<0.001
Hospital days	15 (8; 23)	19 (10; 24)	0.387
HFNC days	3 (2; 4)	3 (2; 3)	0.712
NIMV days	4 (2; 6)	4 (3; 6)	0.936
Albumin (g/dL)	3 (3; 3)	3 (3; 3)	0.072
Prealbumin (mg/dL)	16 (11; 21)	14 (9; 19)	0.351
Retinol (UI)	4 (2; 5)	3 (2; 5)	0.478
Transferrin (md/dL)	158 (122; 178)	145 (122; 168)	0.218
Bilirubin (mg/dL)	0.53 (0.35; 0.70)	0.54 (0.36; 1.08)	0.404
AST (U/L)	36 (21; 56)	38 (24; 48)	0.961
ALT (U/L)	38 (19; 62)	32 (20; 56)	0.612
GGT (U/L)	77 (57; 100)	89 (66; 136)	0.041
ALP(U/L)	69 (40; 142)	66 (36; 120)	0.666
INR	1.10 (1.00; 1.17)	1.15 (1.06; 1.23)	0.026
Prothrombin (s)	13 (12; 14)	14 (13; 25)	0.004
Urea (mg/dL)	52 (38; 71)	55 (40; 78)	0.61
Creatinine (mg/dL)	0.75 (0.60; 1.03)	1.08 (0.76; 1.49)	0.009
Daily data *			
Energy target (Kcal)	1800 (1435; 2100)	1875 (1580; 2492)	0.134
Volume of enteral administration (mL)	457 (250; 500)	408 (309; 500)	0.809
Enteral intake (Kcal)	531 (300; 600)	480 (371; 600)	0.987
Ratio of energy intake/target	0.29 (0.17; 0.38)	0.25 (0.19; 0.33)	0.332
Parenteral dextrose intake (Kcal)	45 (0; 179)	113 (42; 200)	0.013
Prescribed protein (g/day)	50 (50; 94)	50 (50; 50)	0.012
Protein intake (g/day)	50 (25; 50)	40 (24; 50)	0.177
Ratio of protein intake/target	0.83 (0.47; 1.00)	0.80 (0.61; 1.00)	0.397
Gastric residue (mL) (n)	62 (50; 100) (n = 21)	120 (75; 240) (n = 5)	0.103
Total kcal intake (Kcal)	600 (393; 744)	641 (515; 751)	0.247
Caloric intake (Kcal/kg)	7 (5; 10)	7 (6; 10)	0.597
Ratio of total energy intake/target	0.32 (0.22; 0.46)	0.32 (0.25; 0.40)	0.878
PN kcal intake (n)	1518 (1125; 1585) (n = 5)	210 (210; 210) (n = 1)	0.38
PN protein intake (g) (n)	84 (83; 99) (n = 5)	25 (25; 25) (n = 1)	0.143
Propofol kcal intake (n)	82 (52; 112) (n = 2)	75 (38; 112) (n = 2)	1
Glycemia (md/dL)			
Median	130 (111; 152)	130 (119; 159)	0.501
Minimum	110 (94; 127)	116 (92; 130)	0.46
Maximum	154 (130; 182)	152 (132; 206)	0.511

* Daily data for each patient are summarized as medians. Data are presented as means ± SD, frequencies (%), and medians (IQR). Apache: acute physiology and chronic health evaluation. SOFA: sequential organ failure assessment. VAP: ventilator-associated pneumonia. UTIs: urinary tract infections. CRRT: continuous renal replacement therapy. AGDML: acute gastroduodenal mucosal lesions. NG: nasogastric; NGF: nasogastric tube feeding. EN: enteral nutrition. HFNC: high-flow nasal cannulas. NIMV: noninvasive mechanical ventilation. AST: aspartate aminotransferase. ALT: alanine aminotransferase. GGT: gamma glutamyl transpeptidase. ALP: alkaline phosphatase. INR: international normalized ratio. PN: parenteral nutrition.

According to the results of the univariate analysis, the variables included in the multivariate logistic analysis for “*Infectious complications*” were: Apache-II score, SOFA on third day, CRRT, prokinetics, antimicrobial treatments, 90-day mortality, ICU days, INR, prothrombin (s), creatinine (mg/dL), parenteral dextrose intake_Kcal, prescribed protein_g/day.

As shown in Table 5, the variables selected in the multivariate logistic regression were CRRT (OR = 6.054; 95% CI = 1.639–22.37) and ICU days (OR per day = 1.045; 95% CI = 1.013–1.078).

## 4. Discussion

The decision to initiate and manage enteral nutritional therapy, orally or via NGFs, in critically ill patients undergoing oxygen therapy with HFNC and/or NIMV is a topic of debate [30,31,32,33] at present, and it is not adequately addressed in the latest guidelines on nutritional therapy in ICU patients [13,25]. The most recent guidelines advise lowering the recommended prescribed energy to 12–25 kcal/kg/day [13]. Despite the potential benefits associated with this practice, the scarcity of clinical studies of sufficient methodological quality in patients, as well as the absence of specific recommendations on enteral nutritional therapy for this particular type of patient, have generated controversy among professionals involved in the care of the critically ill [22,34,37,38,39].

In our study, we observed that the administration of trophic nutrition in this cohort of critically ill patients, who were well nourished upon admission, and who needed oxygen therapy by means of HFNC or NIMV, seems feasible and safe in patients with hypoxic respiratory failure. Our main objective was to study 90-day mortality, and, after an analysis of the reported data, we observed that, even being a group of critically ill patients with high severity scores and difficult nutritional management, mortality was low (14.8%), as found in other studies [23] on trophic nutrition in ICU patients, showing that the use of trophic or complete feeding in ICU patients, regardless of their nutritional risk, had no effect on clinical outcomes [40,41]. Furthermore, the mortality data from our observational trophic nutrition study are more aligned with the results of Wang CY, et al., who even found a slightly lower in-hospital mortality rate in the trophic feedings group compared to the complete feedings group in a recent randomized–controlled trial [40]. Finally, CRRT, bacteremia, and age were the variables independently associated with survival in our study.

The chosen diet was nutritionally complete, high in protein, based on whey peptides, low in fiber, and had a reduced fat content of 24% of the total energy, in addition to providing 40% of the fat as medium-chain triglycerides with an additional three grams per liter in the form of fish oil. As it is a hyperproteic formula administered at trophic doses, the volume of the diet administered was low, at 450 mL, thus facilitating food tolerance and not interfering with the oxygen therapy modality used with these patients. In addition, a daily median of 50 g of protein was provided. We were also able to observe the patients’ preference for oral nutrition administration (76.9%) over NGFs and the feasibility of this method of administration without increasing the risk of serious side effects.

The observed and described adverse effects caused by the use of this type of nutrition that could compromise patient safety were few and did not lead to any untreatable complications, but rather the interruption of enteral therapy. They caused the interruption of trophic nutrition administration in ten of the thirty patients who presented adverse effects—6.7% of the total number of patients studied. Diarrhea in seven cases and abdominal distension in two were the main factors that were significantly associated with a feeding interruption (*p* < 0.05). Diarrhea is not uncommon and is multifactorial in ICU patients, and the administered diet was rarely its only cause. The rate of diarrhea rate was 12.1% in our study, lower than the 14.7% reported in a seminal study in ICU patients in our country [42].

There are no recommended common guidelines or protocols on how to proceed with oral feedings in ICU patients receiving HFNC oxygen therapy and/or NIMV, particularly for the prevention of malnutrition or refeeding syndrome. The nutritional intakes of these patients should be closely monitored to ensure they are adequate during their stay in the ICU, and total daily fluid administration is of concern, as discussed above. Oral nutritional therapy is more common in nonventilated patients and both parenteral and enteral nutrition in ventilated patients than in spontaneously breathing patients. However, although most of our patients were receiving oral nutrition, we were able to feed them while they were undergoing respiratory therapy. Importantly, there were no significant differences among patients in terms of the need to discontinue trophic feedings according to the route of nutritional therapy administered. The need to discontinue trophic feedings was significantly associated with the SOFA score upon admission and, to a much lesser degree, with plasma urea levels, which have recently been associated with protein administration and worsening outcomes in patients with acute kidney injury and elevated organ failure scores [26].

The administration of oral or enteral nutritional therapy in these patients may be perceived as unsafe because of the possible risk of aspiration. Therefore, most of these patients are often denied adequate caloric and protein intakes. HFNC therapy and NIMV allowed us to provide more adequate nutrition, mainly via the oral route. In this regard, short-lived proteins such as prealbumin and retinol did not vary significantly during our study period, corroborating the appropriateness of trophic enteral therapy in these patients. The amount of nutrient intake administered parenterally in our study was low. This could be due to the fact that most of our patients were awake and cooperative. Only four patients (2.7%) received propofol, with a low average caloric intake, and all of them survived. In addition, six patients, apart from enteral nutrition, also received PN, and five of them survived. We successfully addressed the well-known “eat or breathe” dilemma by feeding our patients and allowing them to both feed and breathe. Indeed, our results show that we achieved both [43].

Despite the severity score values upon admission and on day 3, the rates of relevant infections, such as VAP and tracheobronchitis, were not excessive, perhaps due to the use of trophic rather than complete nutrition, which is better tolerated gastrointestinally, as previously described [41], and most likely contributes to the safety of this nutritional treatment. Of note, there were no significant differences among patients in the development of new infections during trophic feedings according to the route of enteral nutritional therapy administration, although infected patients received significantly more prokinetics versus the noninfected group. Infectious complications were significantly associated with the need to receive CRRT and, to a lesser degree, with the length of ICU stay [44].

The limitations of our study include its observational nature and possible selection bias, which may limit the generalizability of the results. Nevertheless, they provide valuable information on the feasibility, safety, and potential benefits of trophic nutrition in critically ill patients. Future research should focus on randomized–controlled trials to better elucidate the timing, amount, and composition of nutritional support in noninvasively oxygenated or ventilated patients, with particular emphasis on the roles of proteins in improving outcomes. Another limitation of our study is the small subset of participants who received NIMV (11 out of 149), and we suggest caution in applying the present results to NIMV users.

The results of this prospective and observational study open the door to the possibility of administering enteral nutrition, either via NGFs or orally, almost immediately, to a fairly large group of patients, such as critical patients with acute respiratory failure in need of NIMV and/or HFNC therapy, thus advancing the optimization of one of the pillars of critical treatment, such as maintaining intestinal trophism and reducing malnutrition, as well as the resulting complications that occur in these patients [43].

## 5. Conclusions

In conclusion, in our study, we show that the early administration of trophic nutrition, either orally or by nasogastric tube, of a specific hyperproteic diet in patients admitted to the ICU, with acute respiratory failure and without malnutrition, who require oxygen therapy with MNIV or HFNC, is feasible, well tolerated and safe. It is associated with a 90-day mortality similar to or lower than in other studies in critically ill patients receiving enteral trophic nutrition administration.

## Figures and Tables

**Table 2:** Multivariate logistic regression on survival.

Variables *	Coefficient (SE)	*p*-Value **	BIC ***	OddsRatio (95% CI)
(Intercept)	−6.447 (1.649)	-	-	-
Age, per year	0.056 (0.023)	0.007	119.2	1.057 (1.011; 1.106)
Bacteremia	2.183 (0.763)	0.004	120	8.869 (1.990; 39.531)
CRRT	2.100 (0.752)	0.006	119.5	8.168 (1.871; 35.667)

CRRT: continuous renal replacement therapy. BIC: Bayesian information criterion. * Variables were selected using the best subset regression procedure. ** Likelihood ratio test. *** BIC, if the variable was dropped. The BIC is a measure of the lack of fit of the model, and the BIC of the full model was 120.9. Note that if any variable is removed, the BIC of the model increases (i.e., a worse model).

**Table 3:** Multivariate logistic regression on the discontinuation of nutritional therapy via oral feedings/NGFs.

Variables *	Coefficient (SE)	*p*-Value **	BIC ***	Odds Ratio (95% CI)
(Intercept)	−7.956 (1.785)	-	-	-
SOFA upon admission, per unit	0.379 (0.168)	0.016	49.7	1.461 (1.051; 2.032)
Urea, per mg/dL	0.029 (0.008)	<0.001	61.8	1.029 (1.013; 1.045)

SOFA: sequential organ failure assessment. BIC: Bayesian information criterion. * Variables were selected using the best subset regression procedure. ** Likelihood ratio test. *** BIC, if the variable was dropped. The BIC is a measure of the lack of fit of a model. The BIC of the full model was 48.7. Note that if any variable is removed, the BIC of the model increases (i.e., worse model).

**Table 5:** Multivariate logistic regression on infectious complications.

Variables *	Coefficient (SE)	*p*-Value **	BIC ***	Odds Ratio (95% CI)
(Intercept)	−1.872 (0.321)	-	-	-
CRRT	1.801 (0.667)	0.005	157.9	6.054 (1.639; 22.37)
ICU days (per day)	0.044 (0.016)	0.004	158.6	1.045 (1.013; 1.078)

CRRT: continuous renal replacement therapy. ICU: intensive care unit. BIC: Bayesian information criterion. * Variables were selected using the best subset regression procedure. ** Likelihood ratio test. *** BIC, if the variable was dropped. The BIC is a measure of the lack of fit of a model. The BIC of the full model was 155. Note that if any variable is removed, the BIC of the model increases (i.e., worse model).

## Data Availability

Data are only available upon request due to institutional restrictions.

## Supplementary Materials

The following supporting information can be downloaded at: https://www.mdpi.com/article/10.3390/nu16091366/s1. Table S1: Patient characteristics according to the need to discontinue nutritional therapy.
