# Supplementary material for: Trophic Nutrition in ICU Patients Undergoing High-Flow Oxygen Therapy and/or Noninvasive Mechanical Ventilation: The Nutri-Trophic Study
Source: Nutrients. 2024 Apr 30;16(9):1366. doi: 10.3390/nu16091366 (PMC11085204; doi:10.3390/nu16091366)
Supplement: Supplementary file 1 [file nutrients-16-01366-s001.zip › nutrients-2966501-supplementary.pdf]

Supplementary Materials: The following supporting information can be downloaded at: www.mdpi.com/xxx/s1, Table S1: Patient characteristics according to the need to discontinue nutritional therapy.

Table S1. Patient characteristics according to the need to discontinue nutritional therapy.

		Need to discontinue nutritional therapy		
		No<br>N = 139	Yes<br>N = 10	p-Value
Age (years)		62.5 ± 13.7	64.0 ± 17.9	0.749
Sex male		96 (69.1)	6 (60.0)	0.726
Body mass index (kg/m^2)		28.0 ± 5.6	29.6 ± 5.1	0.387
Apache-II score		14 (10; 19)	22 (16; 26)	0.007
SOFA upon admission		3 (2; 6)	8 (7; 9)	< 0.001
SOFA on day 3		3 (2; 5)	6 (5; 7)	0.002
Complications				
Tracheobronchitis		12 (8.6)	0	1
VAP		10 (7.2)	0	1
Bacteremia		12 (8.6)	0	1
UTIs		9 (6.5)	0	1
Other infections		8 (5.8)	1 (10.0)	0.474
CRRT		12 (8.6)	0	1
Prophylaxis_AGDML		122 (87.8)	7 (70.0)	0.134
Prokinetics		21 (15.1)	1 (10.0)	1
Antimicrobial treatments		75 (54.0)	6 (60.0)	0.755
90-Day mortality		19 (13.7)	3 (30.0)	0.168
Oral feedings		101 (75.9)	9 (90.0)	0.454
NGFs		35 (26.3)	1 (10.0)	0.452
Diarrhea		11 (7.9)	7 (70.0)	< 0.001
Gastric residue > 500 ml (n)		0	2 (20.0)	0.004
Vomiting/regurgitation		1 (0.7)	0	1
Broncho-aspiration		0	0	1
NG tube obstruction		1 (0.7)	0	1
Abdominal distention		2 (1.4)	2 (20.0)	0.023
NG tube displacement		4 (2.9)	0	1
EN discontinuation		0	10 (100.0)	< 0.001
Oxygen therapy type:				1
HFNC		128 (92.1)	10 (100.0)	
NIMV		11 (7.9)	0	
ICU days		9 (6; 15)	10 (8; 18)	0.267
Hospital days		14 (8; 23)	24 (22; 25)	0.002
HFNC days		3 (2; 4)	4 (2; 8)	0.28
NIMV days		4 (2; 6)	5 (3; 8)	0.17
Albumin (g/dL)		3 (3; 3)	3 (3; 3)	0.463
Prealbumin (mg/dL)		16 (10; 21)	12 (8; 15)	0.2
Retinol (UI)		4 (2; 5)	4 (4; 6)	0.229
Transferrin (md/dL)		155 (123; 176)	117 (103; 149)	0.2
Bilirubin (mg/dL)		0.52 (0.35; 0.70)	1.12 (0.43; 1.57)	0.03
AST (U/L)		38 (22; 53)	22 (20; 64)	0.489
ALT (U/L)		38 (20; 60)	20 (15; 43)	0.117

		Need to discontinue nutritional therapy		
		No<br>N = 139	Yes<br>N = 10	p-Value
GGT (U/L)		79 (59; 102)	130 (73; 167)	0.085
ALP(U/L)		66 (38; 137)	131 (79; 433)	0.018
INR		1.10 (1.01; 1.18)	1.12 (1.05; 1.20)	0.537
Prothrombin (sec)		13 (12; 15)	13 (13; 21)	0.338
Urea (mg/dL)		50 (38; 69)	144 (101; 201)	< 0.001
Creatinine (mg/dL)		0.79 (0.61; 1.10)	1.54 (0.91; 2.53)	0.013
Daily data *				
Energy target (Kcal)		1811 (1500; 2125)	2075 (1488; 2422)	0.489
Volume of enteral administration (ml)		450 (288; 500)	265 (142; 546)	0.223
Enteral intake (Kcal)		531 (302; 600)	318 (131; 656)	0.297
Ratio of energy intake / target		0.28 (0.19; 0.36)	0.24 (0.07; 0.37)	0.358
Parenteral dextrose intake (Kcal)		76 (0; 200)	33 (1; 54)	0.23
Prescribed protein (g/day)		50 (50; 90)	82 (45; 119)	0.212
Protein intake (g/day)		48 (25; 50)	74 (13; 96)	0.263
Ratio of protein intake / target		0.80 (0.49; 1.00)	0.93 (0.55; 1.00)	0.837
Gastric residue (ml)<br>(n)		62 (50; 125)<br>(n=23)	75 (62; 112)<br>(n=3)	0.717
Total kcal intake (Kcal)		600 (460; 733)	849 (184; 1137)	0.378
Caloric intake (Kcal / kg)		7 (5; 10)	9 (3; 14)	0.59
Ratio of total energy intake / target		0.31 (0.23; 0.43)	0.62 (0.12; 0.81)	0.297
PN kcal intake<br>(n)		1518 (864; 1552) (n=3)	1125 (642; 1659) (n=3)	0.827
PN protein intake (g)<br>(n)		74 (50; 87)<br>(n=3)	84 (84; 93)<br>(n=3)	0.275
Propofol kcal intake<br>(n)		82 (18; 143)<br>(n=4)	(n=0)	
Glycemia (mg/dL)				
Median		129 (111; 153)	140 (134; 154)	0.306
Minimum		111 (92; 128)	104 (96; 128)	0.955
Maximum		152 (127; 190)	164 (149; 178)	0.359

(*) Daily data for each patient are summarized as medians. Data are presented as the means ± SD, frequencies (%), and medians (IQR). Apache: acute physiology and chronic health evaluation; SOFA: sequential organ failure assessment; VAP: ventilator-associated pneumonia; UTIs: urinary tract infections; CRRT: continuous renal replacement therapy; AGDML: acute gastroduodenal mucosal lesions; NG: nasogastric; NGF: nasogastric tube feeding; EN: enteral nutrition; HFNC: high-flow nasal cannulas; NIMV: noninvasive mechanical ventilation; AST: aspartate aminotransferase; ALT: alanine aminoytransferase; GGT: gamma glutamyl transpeptidase; ALP: alkaline phosphatase; INR: international normalized ratio; PN: parenteral nutrition.

The median estimated energy expenditure was 1811 kcal, and there were no significant differences among the groups studied. The median amount of protein administered was 45 g/day, with significantly higher values for patients who had no interruptions in trophic enteral therapy ($p<0.05$). Gastric residual was negligible in both studied groups, with a median of 69 ml, and only two patients had residual values greater than 500 ml.

The median amount of daily kilocalories administered to patients via trophic feedings was 520 ml, the median protein intake was 50 g, and there were no significant differences between patients who did and did not experience interruptions in the trophic feedings. There were also no significant differences between BMI, albumin, prealbumin, retinol, transferrin, and liver enzymes among the groups studied.

One hundred and ten patients received oral trophic feedings, thirty-six patients received nasogastric tube feedings, and three mixed feedings. There were no significant differences in required interruptions of enteral nutrition administration. Table 3 also shows the daily data for the amounts of both prescribed and administered calories and protein, as well as the ratio of prescribed to administered. There were also no statistically significant differences among the groups studied. Propofol was administered to four patients (median kcal: 82 ml), none of whom required interruptions in enteral nutrition administration. In addition, six patients received parenteral nutrition, three of whom required an interruption in enteral nutrition administration.
